# Psychotropic prescribing patterns during pregnancy in two South African mental health clinics

**DOI:** 10.4102/jcmsa.v3i1.188

**Published:** 2025-10-17

**Authors:** Catherine Farmer, Elsa du Toit, Ulla Botha, Dana Niehaus, Liezl Koen

**Affiliations:** 1 Department of Psychiatry, Faculty of Medicine and Health Sciences, Stellenbosch University, Cape Town, South Africa; 2 Panorama Healthcare Psychiatry, Cape Town, South Africa

**Keywords:** psychotropics, pregnancy, polypharmacy, maternal mental health, prescription patterns, psychiatric diagnosis

## Abstract

**Background:**

Given that there is a significant burden of mental illness during pregnancy, psychotropic polypharmacy during this period is commonly found in clinical practice. In South Africa, however, there is a paucity of data on the use of psychotropics during pregnancy.

**Methods:**

This was a retrospective descriptive study of 303 pregnant women attending two specialised maternal mental health clinics from presentation to six weeks postpartum. Demographic data, psychiatric history, medical comorbidity, pregnancy-related, and prescription data were collected and tabulated at treatment-as-usual visits. Polypharmacy prevalence was defined as the prescription of two or more psychotropics for at least 90 days.

**Results:**

A majority of the study group was diagnosed with major depressive disorder (*n* = 161, 53.1%), and non-tricyclic antidepressants were the most prescribed medication class (*n* = 195, 64.4%). One-third of the participants received prescriptions in all three trimesters. Polypharmacy criteria were met in 18.8% (*n* = 57) of the sample population.

**Conclusion:**

The prescription patterns in the study sample appeared to be in line with current international protocols. Prescribing psychotropics during pregnancy remains challenging because of the need to weigh up the potential risks of medication-related effects on the mother and baby against those of discontinuing treatment.

**Contribution:**

This study may raise awareness and assist medical professionals regarding the rational use of psychotropic medication during pregnancy.

## Introduction

Pregnancy is associated with a significantly increased risk for new onset or relapse of mental illness, both in high and low- to middle-income countries (LMICs).^1,2^ In Northern Ireland, Mongan et al.^3^ reported a prevalence of 18.9% for self-reported mental disorders during pregnancy. In LMICs, this prevalence appears to be higher with Faisal-Cury et al.^4^ reporting 20.2% in Sao Paulo, Brazil and Woldetadik et al.^5^ reporting 35.8% in South-East Ethiopia. In South Africa, Zar et al.^6^ conducted a study in pregnant women of black African and mixed ancestry, attending two public health facilities in the Drakenstein area in Paarl, Western Cape province. Pregnant women were enrolled in their second trimester and followed through childbirth. The study reported that 24% of pregnant women scored above the threshold for depression using the Edinburgh Postnatal Depression Scale (EPDS) and 20% scored above the threshold for psychological distress using the Self-Reporting Questionnaire (SRQ-20). Hartley et al.^7^ also reported depressed mood (EPDS score-based) in 39% of mothers in a community-based, cluster-randomised controlled trial in Khayelitsha and Mfuleni in Cape Town, South Africa.

Failure to treat mental illness during pregnancy could result in negative outcomes for both the mother and the baby. A systematic review found that maternal mental illness increased the risk for infant mortality and/or stillbirth,^8^ and that the risk of having a preterm birth or delivering a baby with low birth weight was higher for women with untreated depression.^9^ Further, the impact of maternal mental illness during pregnancy may also extend to later developmental periods of the baby, where adolescents who were exposed to maternal depression *in utero* and during the postnatal period were reported to have a 4.7 times greater risk of developing depression compared to unexposed babies.^10^ For mothers, the strongest determinant of postnatal depression is antenatal depression.^11^

Potential risks of psychotropic medication use during pregnancy can be divided into two broad categories.^12^ Firstly, risks to the developing child, including teratogenicity, withdrawal and long-term behavioural effects and secondly, pregnancy complications that could affect both the mother and the baby, including spontaneous abortion, pre-eclampsia and postpartum haemorrhage. The management of psychotropic medications in women planning pregnancy and those already pregnant requires careful risk-benefit assessment, as all psychotropic medications cross the placenta and are present in amniotic fluid.^13,14^ Current guidelines^15,16,17,18^ emphasise that the safest and most effective maternal dose should be prescribed when the benefits of treatment outweigh potential risks. Administering higher doses of a single pharmacological agent is generally preferable to polypharmacy. Additionally, the selection of drugs characterised by limited metabolite formation, high protein-binding affinity and a low potential for drug–drug interactions is recommended. Pre-conception counselling is emphasised as a critical component of care, with National Institute for Health and Care Excellence (NICE) guidelines specifically addressing the recognition, assessment and treatment of mental health problems in women planning pregnancy.^19^ The South African Department of Health’s Standard Treatment Guidelines and Essential Medicines List for South Africa also provide prescribing guidelines for women of childbearing age and during pregnancy.^17,18^

For antidepressants, selective serotonin reuptake inhibitors remain first-line treatments and are considered safe to use during pregnancy when treatment is needed, although paroxetine should be avoided across all trimesters and benzodiazepines should be avoided during the first trimester because of teratogenic risks and potential neonatal withdrawal symptoms.^20,21,22,23^ Regarding antipsychotics, several recent reviews have shown that the majority of studies have found antipsychotic use during pregnancy to be safe, with risperidone being a possible exception.^24,25^ It is thus recommended that women who need to take antipsychotics continue to do so during pregnancy. While no currently available antidepressants or antipsychotics are contraindicated during pregnancy, healthcare providers should consider the risks and benefits of the antipsychotics and antidepressants prescribed.^24,26^ Mood stabilisers (i.e. some antiseizure medications are used as mood stabilisers for the management of bipolar disorder), such as lamotrigine and lithium, are considered acceptable options with appropriate risk-benefit analysis,^27,28^ while the use of lithium during the first trimester has been shown to increase the risk for major malformations.^29^ The use of other mood stabilisers, especially sodium valproate and carbamazepine, should be avoided because of the high risk of major malformations and long-term developmental defects.^30,31,32^

Discontinuation of medication in pregnant women diagnosed with a serious mental illness could pose significant risks. Seki et al.^33^ reported a relapse rate of 16.7% in a group of pregnant women with schizophrenia who remained on treatment versus 90.0% in the discontinuation group. This increased risk seems to cross diagnostic borders, where women with bipolar disorder who discontinued their medication versus those who continued mood stabiliser treatment had a two-fold increased recurrence risk, a shorter time to recurrence and spent more time being ill during their pregnancy.^34^ According to Wesseloo et al.,^35^ patients with a diagnosis of bipolar mood disorder not receiving prophylactic medication during pregnancy had significantly higher rates of relapse during the postpartum period compared to those on prophylactic treatment (66% [95% confidence interval: 57–75] vs. 23% [95% confidence interval: 14–37]).

The ideal approach to managing pregnant patients with mental illness is through pregnancy planning and individualising treatments based on the risk profile of the patient.^36^ However, the prevalence of unintended or unplanned pregnancy in sub-Saharan Africa averages about 29%.^37^ In South Africa, Du Toit et al.^38^ reported that more than half of the patients presented with an unplanned pregnancy. It therefore becomes necessary to focus on a pragmatic risk-based management plan where the foetal risks of continued exposure to potentially dangerous drugs should be carefully weighed up against the risk of relapse for the mother if medication were to be discontinued.^36,39^

There is a paucity of data on the use of psychotropic medication during pregnancy in South African women suffering from a mental illness. Using data from a previous maternal mental health outcomes umbrella study,^38^ this study aimed to describe the psychotropic medication prescription patterns and prevalence of polypharmacy during pregnancy at specialist-level maternal mental health clinics in the Western Cape province of South Africa.

## Research methods and design

### Study design

This was a descriptive, retrospective cohort study of psychotropic medication prescriptions in 303 pregnant women attending two secondary-level specialist maternal mental health clinics in Cape Town, South Africa. Participants were included from conception until six weeks postpartum.

### Study setting

The umbrella study^38^ was conducted at two specialised maternal mental health clinics in Cape Town, South Africa – one private sector (Sophia Perinatal Psychiatry Clinic) and one state sector (Stikland Maternal Mental Health Outpatient Clinic). Stikland Hospital is a public tertiary-level stand-alone psychiatric hospital operating in the Northern and Tygerberg Districts of Cape Town. The hospital provides all levels of psychiatric care to a part of the Tygerberg East metro region, as well as to the West Coast and Winelands rural regions. The Sofia Perinatal Psychiatry Clinic, a division of the Panorama Healthcare Psychiatry, is a privately owned clinic operating in the Northern District of Cape Town. Both clinics provide outpatient perinatal mental healthcare. Patients accessing care at the private Sofia clinic typically earn more than those accessing care from the state clinic, as they require either medical insurance coverage or sufficient personal finances to pay for private healthcare services. Patients were mostly referred to the Stikland and Sophia clinics by general physicians working in primary healthcare, but patients seen at the Sophia clinic could also be self-referred.

### Study sample

Participants included in this study formed part of a larger study that aimed to describe the maternal and foetal outcomes of pregnancy in women with mental illness in a developing country and to identify risk factors for adverse pregnancy outcomes.^38^ The larger study used convenience sampling for participant recruitment, through enrolling (after receiving voluntary written, informed consent) participants who accessed mental healthcare services at one of the specialist clinics. Females (aged 18–45 years) with a primary Diagnostic and Statistical Manual of Mental Disorders, fourth edition, text revision (DSM-IV-TR) diagnosis of severe psychiatric illness, including psychotic, mood and anxiety disorders, were included. A recent general household survey by Statistics South Africa in 2018^40^ determined that 71.5% of South Africans had access to public healthcare, while 27.1% had access to private healthcare. The umbrella study participants were recruited in a ratio of 3:2 from the state and private clinics, respectively. The ratio of recruitment was chosen to give a true-to-life picture of the current pattern of referral to specialist psychiatric services.

### Data collection

Data were collected during the four care-as-usual visits (an initial evaluation followed by one visit per trimester and six weeks postpartum) and captured onto an Excel spreadsheet (umbrella study). The current study expanded the database by capturing detailed information on the psychotropic medication prescriptions using the existing case report forms and clinical case notes (including prescription charts).

Demographic data, psychiatric history, medical comorbid illnesses, as well as pregnancy-related data were tabulated. Medication received by the participants was divided into 12 categories (Table 1). Because of their dissimilar side effect profiles, different classes of antipsychotics, antidepressants and mood stabilisers were analysed separately. Polypharmacy was defined as the use of two or more psychotropic medication prescriptions for at least 90 days, as recommended in the validation study by Leckman-Westin et al.^41^ and used in a similar study by Broeks et al.^42^ If there was a medication change, then this was seen as a new polypharmacy episode.

**TABLE 1:** Types of medications received by study participants.

Category	Drug class	Specific medications
1	Non-tricyclic antidepressants	Fluoxetine Citalopram Venlafaxine
2	Tricyclic antidepressants	Amitriptyline
3	Typical antipsychotics	Haloperidol Chlorpromazine
4	Atypical antipsychotics	Aripiprazole Olanzapine Clozapine Risperidone
5	Mood stabilisers†	Sodium valproate
6	Mood stabilisers†	Lamotrigine
7	Mood stabilisers†	Carbamazepine
8	Mood stabilisers†	Topiramate
9	Mood stabilisers†	Lithium carbonate
10	Analgesic and anticonvulsant gamma-aminobutyric acid (GABA)	Gabapentin – sometimes used off-label in psychiatry for anxiety and other disorders
11	Sedative hypnotics	Benzodiazepines Non-diazepine hypnotics
12	Other medications	Beta-blockers Anticholinergics Antihistamines

† , considered separately because of their distinct safety profiles.

### Data analysis

Data were analysed using IBM SPSS Statistics, Version 28 ([computer program]. Armonk, NY: IBM Corporation; 2021). Continuous variables were summarised as mean and standard deviation or median and interquartile ranges, while nominal variables were summarised as counts and percentages. Because this was a descriptive study, no further statistical methods were employed.

### Ethical considerations

Ethical clearance to conduct this study was obtained from the Health Research Ethics Committee of Stellenbosch University (Umbrella Study HREC reference number N12/10/071; current Study HREC reference number S21/04/063) and the National Department of Health. Stringent protocols were put in place to ensure the safety and confidentiality of patient records during the data collection phase, and all collected data were anonymised by the removal of direct identifiers. A waiver of informed consent was granted, given the retrospective nature of the study. This study was conducted in accordance with the Declaration of Helsinki.^43^

## Results

### Demographic variables

The study sample comprised 303 participants with a mean age of 31.0 years (s.d. = 5.51) and a mean paternal age of 34.8 years (s.d. = 6.62). Participants were mostly married (*n* = 178, 58.7%), 48.8% (*n* = 148) were employed, 39.9% (*n* = 121) had a tertiary-level education, and Afrikaans was the most commonly spoken language (*n* = 212, 70.0%) (Table 2).

**TABLE 2:** Demographic characteristics of the study sample (*N* = 303).

Variable	*n*	%
**Marital status**		
Married	178	58.7
Unmarried	125	41.2
**Employment**		
Employed	148	48.8
Unemployed	129	42.5
Disability grant	24	7.9
Student	1	0.3
Missing†	1	0.3
**Highest level of education**		
Primary school	19	6.3
Grade 8–10	51	16.8
Grade 11–12	111	36.6
Tertiary education	121	39.9
Missing†	1	0.3
**Language**		
Afrikaans	212	70.0
English	65	21.5
isiXhosa	21	6.9
French	3	1.0
English and Afrikaans	1	0.3
Missing†	1	0.3

*Source:* Adapted from Farmer C. Psychotropic medication prescribing patterns during pregnancy in two South African Maternal Mental Health clinics [MMed thesis]. Stellenbosch: Stellenbosch University; 2023

† , Refers to multiple causes for missing data.

### Pregnancy-related variables

The study participants’ initial visit to the treatment facilities spanned all three trimesters, where 43.6% (*n* = 132) presented in their first trimester, 38.3% (*n* = 116) presented in their second trimester and 16.8% (*n* = 51) presented in their third trimester. The mean gestational age at the initial visit was 17.0 weeks (s.d. = 9.14, range = 36). Gravidity ranged from one (*n* = 80, 26.4%) to eight (*n* = 1, 0.3%), with the majority having had two pregnancies (*n* = 93, 30.7%), while parity ranged from zero (*n* = 112, 37.0%) to four (*n* = 6, 2.0%), with the majority having had given birth to one foetus with a gestational age of 24 weeks (*n* = 115, 38.0%). Most participants had not miscarried (*n* = 213, 70.3%) or terminated their pregnancies (*n* = 266, 87.8%). More than half reported that their pregnancies were planned (*n* = 159, 52.5%), 69.3% (*n* = 210) indicated that they were anticipating a normal vertex delivery and 80.2% (*n* = 243) planned to breastfeed.

### Medical comorbidity

Almost a quarter (*n* = 73, 24.1%) of participants reported comorbid medical illnesses, with endocrine (*n* = 15, 5.0%) and cardiac (*n* = 15, 5.0%) disorders being the most common (Table 3). Injuries and acute infections were not included in this analysis.

**TABLE 3:** The prevalence of medical disorders (system-based) in the study participants (*N* = 303).

Medical disorders	*n*	%
Endocrine	15	5.0
Cardiac	15	5.0
Pregnancy related	10	3.3
Respiratory	10	3.3
Infectious diseases	8	2.6
Neurological	8	2.6
Haematological	4	1.3
Dermatological	3	1.0
Rheumatological	3	1.0
Gastroenterological	2	0.7
Gynaecological	2	0.7
Otorhinolaryngological	2	0.7
Renal	2	0.7
**Number of comorbid disorders**		
No medical illness	230	75.9
1 medical illness	57	18.8
> 1 medical illness	16	5.3

*Source:* Adapted from Farmer C. Psychotropic medication prescribing patterns during pregnancy in two South African Maternal Mental Health clinics [MMed thesis]. Stellenbosch: Stellenbosch University; 2023

### Psychiatric disorders

Major depressive disorder was the most prevalent (*n* = 161, 53.1%), followed by bipolar I disorder (*n* = 41, 13.5%) and schizophrenia (*n* = 37, 12.2%) (Table 4). Most participants had one psychiatric diagnosis (*n* = 217, 71.6%). Almost half of the participants had a psychiatric admission (*n* = 138, 46.0%), with a median of one psychiatric admission (IQR = 1–2, range = 1–9). The mean length of psychiatric admission was 13.01 days (s.d. = 27.95) for those who were admitted. The mean number of suicide attempts was 0.50 (s.d. = 0.76).

**TABLE 4:** Participants’ psychiatric disorders according to diagnostic and statistical manual of mental disorders, fourth edition, text revision.

Psychiatric disorders	*n*	%
**Attention-deficit/hyperactivity disorder**	1	0.3
**Schizophrenia and other psychotic disorders**		
Schizophrenia	37	12.2
Schizoaffective disorder	7	2.3
Substance and/or medication-induced psychotic disorder	2	0.7
Unspecified schizophrenia spectrum and other psychotic disorder	2	0.7
**Mood disorders: Bipolar disorders**		
Bipolar I disorder	41	13.5
Bipolar II disorder	20	6.6
Unspecified bipolar and related disorders	1	0.3
**Mood disorders: Depressive disorders**		
Major depressive disorder	161	53.1
Major depressive disorder with psychotic features	8	2.6
Major depressive disorder unspecified	1	0.3
**Anxiety disorders**		
Specific phobia	1	0.3
Panic disorder	14	4.6
Generalised anxiety disorder	28	9.2
Unspecified anxiety disorder	1	0.3
**Anxiety disorders: Obsessive-compulsive and related disorders**		
Obsessive-compulsive disorder	10	3.3
**Anxiety disorders: Trauma- and stressor-related disorders**		
Post-traumatic stress disorder	10	3.2
Adjustment disorder	3	1.0
**Somatoform disorders**		
Conversion disorder	1	0.3
**Eating disorders**		
Bulimia nervosa	2	0.7
**Substance-related disorders**		
Alcohol-related disorder	5	1.7
Cannabis-related disorder	2	0.7
Sedative-, hypnotic- or **anxiolytic**-related disorder	3	1.0
**Stimulant-related disorder**		
**Cocaine**-related disorder	1	0.3
**Methamphetamine**-related disorder	7	2.3
More than one substance	9	3.0
**Delirium, dementia and amnestic and other cognitive disorders**		
Major or mild neurocognitive disorder because of HIV infection	1	0.3
Unspecified neurocognitive disorder	1	0.3
**V-Code**	1	0.3
**Number of psychiatric diagnoses**		
1 disorder†	217	71.6
2 disorders	83	27.4
≥ 3 disorders	3	1.0

Source: Adapted from Farmer C. Psychotropic medication prescribing patterns during pregnancy in two South African Maternal Mental Health clinics [MMed thesis]. Stellenbosch: Stellenbosch University; 2023

One patient had both substance-induced psychotic disorder and schizophrenia listed as their diagnosis, which was amended to ‘Substance Induced Psychotic Disorder’ based on a review of the diagnostic criteria

HIV, human immunodeficiency virus.

† , Patients using more than one substance were counted as having one disorder.

### Substance use history during current pregnancy

Most participants reported no alcohol (*n* = 249, 82.1%) or caffeine (*n* = 173, 57.1%) use (Table 5). Participants reported substance use as follows: alcohol use (*n* = 51, 16.8%), methamphetamine use (*n* = 18, 5.9%), cannabis use (*n* = 12, 4.0%) and tobacco use (*n* = 105, 34.7%). Of those using tobacco cigarettes, on average, 3.6 (s.d. = 4.26) cigarettes were smoked per day.

**TABLE 5:** Participants’ substance use history during their current pregnancy.

Substance	*n*	%
**Alcohol**		
Socially	29	9.6
Occasionally	14	4.6
Weekends	8	2.6
None	249	82.1
Missing†	3	1.0
**Caffeine (cups per day)**		
None	173	57.1
≤ 5	100	33.0
> 5 (max 10)	14	4.6
Missing†	16	5.3
**Tobacco cigarettes (number per day)**		
None	198	65.3
≤ 10	79	26.1
> 10	26	8.6
**Cannabis**		
Yes	12	4.0
No	290	95.7
Missing†	1	0.3
**Methamphetamine**		
Yes	18	5.9
No	282	93.1
Missing†	3	1.0

*Source:* Adapted from Farmer C. Psychotropic medication prescribing patterns during pregnancy in two South African Maternal Mental Health clinics [MMed thesis]. Stellenbosch: Stellenbosch University; 2023

† , Refers to multiple causes for missing data.

### Medication prescription patterns by trimester and drug class

The majority of participants (*n* = 289, 95.4%) were on prescribed psychotropics at any point during their pregnancy (Table 6). Of those prescribed psychotropics, just over a third of participants (*n* = 103, 34.0%) were prescribed three psychotropics at any point during their pregnancy. Approximately 20% of participants were prescribed five or more psychotropics during their pregnancy, with one participant prescribed 11 psychotropics throughout their pregnancy. The number of participants on psychotropics decreased by trimester, with 80.9% (*n* = 245) prescribed psychotropics during the first trimester compared to 77.2% (*n* = 2.4) and 66.7% (*n* = 202) for the second and third trimesters, respectively. Per trimester, approximately half of the participants were prescribed one psychotropic. For the total sample, 953 psychotropic items were prescribed.

**TABLE 6:** Number of participants who had psychotropic medication prescriptions by trimester.

Variable	Trimester 1		Trimester 2		Trimester 3		Trimester 1 + 2 + 3	
	*n*	%	*n*	%	*n*	%	*n*	%
**Prescribed a psychotropic**								
No	58	19.1	69	22.8	101	33.3	14	4.6
Yes	245	80.9	234	77.2	202	66.7	289	95.4
**Number of psychotropics prescribed to those on medication**								
1	150	49.5	157	51.8	146	48.2	46	15.2
2	77	25.4	64	21.1	49	16.2	48	15.8
3	14	4.6	13	4.3	5	1.7	103	34.0
4	4	1.3	-	-	2	0.7	33	10.9
≥ 5†	-	-	-	-	-	-	59	19.5

**Total number of medications prescribed**	**362**	**-**	**324**	**-**	**267**	**-**	**953**	**-**

† , 5 psychotropics: *n* = 14, 6 psychotropics: *n* = 28, 7 psychotropics: *n* = 9, 8 psychotropics: *n* = 5, 9 psychotropics: *n* = 2, 11 psychotropics: *n* = 1.

For the specific psychotropics, two-thirds of the participants were prescribed an antidepressant during pregnancy, of which 64.4% (*n* = 195) were prescribed a non-tricyclic and 2% (*n* = 6) a tricyclic antidepressant, where the non-tricyclic antidepressants were prescribed during all trimesters (Table 7). For antipsychotic medications, 13.5% (*n* = 41) were prescribed typical and 29.0% (*n* = 88) were prescribed atypical antipsychotic medications during their pregnancy. Atypical antipsychotics were prescribed during all trimesters. Out of the mood stabilisers, 7.3% (*n* = 22) were prescribed sodium valproate, 8.3% (*n* = 25) were prescribed lamotrigine, 1.7% (*n* = 5) were prescribed carbamazepine, 0.3% (*n* = 1) were prescribed topiramate and 5.3% (*n* = 16) were prescribed lithium during their pregnancies. Furthermore, all sodium valproate, carbamazepine and topiramate were prescribed during the first trimester. Gabapentin was prescribed for one patient during the second trimester of pregnancy. Thirty-four participants (11.2%) were prescribed sedatives during pregnancy. The 19 participants (6.3%) who received other medication (beta-blockers, antihistamine medication and anticholinergic medication, which may have some effect on psychiatric disorders) had it prescribed during the first trimester. Fifty-seven participants (18.8%) met the criteria for psychotropic polypharmacy, which was defined as the use of more than one psychotropic drug for at least 90 days (data not shown).

**TABLE 7:** Number of participants who had psychotropic medication prescriptions by trimester and drug class.

Trimester(s) medications were prescribed	Antidepressants				Antipsychotics				Mood stabilisers										Gabapentin		Sedative hypnotics		Other prescription drugs	
	Non-tricyclic		Tricyclic		Typical		Atypical		Sodium valproate		Lamotrigine		Carbamazepine		Topiramate		Lithium							
	*N*	%	*N*	%	*N*	%	*N*	%	*N*	%	*N*	%	*N*	%	*N*	%	*N*	%	*N*	%	*N*	%	*N*	%
First only	18	5.9	4	1.3	12	4.0	4	1.3	11	3.6	2	0.7	3	1.0	0	-	3	1.0	0	-	11	3.6	11	3.6
Second only	10	3.3	0	-	1	0.3	5	1.7	0	-	3	1.0	0	-	0	-	1	0.3	1	0.3	3	1.0	0	-
Third only	24	7.9	0	-	1	0.3	10	3.3	0	-	2	0.7	0	-	0	-	1	0.3	0	-	6	2.0	0	-
First + Second only	29	9.6	1	0.3	13	4.3	14	4.6	7	2.3	4	1.3	1	0.3	1	0.3	3	1.0	0	-	3	1.0	5	1.7
First + Third only	1	0.3	0	-	0	-	0	-	1	0.3	0	-	0	-	0	-	0	-	0	-	1	0.3	0	-
Second + Third only	11	3.6	0	-	1	0.3	6	2.0	0	-	1	0.3	0	-	0	-	0	-	0	-	1	0.3	0	-
First + Second + Third	102	33.7	1	0.3	13	4.3	49	16.2	3	1.0	13	4.3	1	0.3	0	-	8	2.6	0	-	6	2.0	3	1.0

**Total**	**195**	**64.4**	**6**	**2.0**	**41**	**13.5**	**88**	**29.0**	**22**	**7.3**	**25**	**8.3**	**5**	**1.7**	**1**	**0.3**	**16**	**5.3**	**1**	**0.3**	**34**	**11.2**	**19**	**6.3**

*Source:* Adapted from Farmer C. Psychotropic medication prescribing patterns during pregnancy in two South African Maternal Mental Health clinics [MMed thesis]. Stellenbosch: Stellenbosch University; 2023

## Discussion

This study sought to describe the psychotropic medication prescription patterns and rates of polypharmacy during pregnancy in patients attending two maternal mental health clinics in Cape Town, South Africa. Overall, antidepressants were the most prescribed class of medication, and just under a fifth of the participants met the criteria for polypharmacy during pregnancy.

In our study, the antidepressant prescription rate aligns with major depressive disorder being the most prevalent diagnosis in our sample. Evidence for the risk of cardiac abnormalities in the offspring of women using antidepressants during pregnancy is mixed. A study by Knudsen et al.,^44^ investigating all registered pregnancies (*n* = 72 280) in Funen, Denmark, from 1995 to 2008, reported a fourfold increased risk for cardiac abnormalities during the first trimester. However, other studies have concluded that the offspring of patients using selective serotonin reuptake inhibitors (SSRIs) did not show an increased risk of major cardiac abnormalities.^45,46^ With regard to tricyclic antidepressants, Gentile^47^ noted an increased risk for congenital malformations with tricyclic antidepressant exposure during the first trimester. It was also noted that when tricyclic antidepressants were used late in pregnancy, the maternal risks included a possible increased risk of pre-eclampsia and a mild popliteal artery entrapment syndrome. The low usage of this type of antidepressant in our sample is in keeping with research data on the safety of these drugs.

Antipsychotic medications were the second most common drug prescribed in our patient sample. In a study conducted in the USA, based on the nationwide Medicaid Analytic Extract database (01 January 2000 to 31 December 2010), the use of both atypical and typical antipsychotics was not significantly associated with an increased risk for congenital malformations overall or cardiac malformations in particular.^48^ The overall use of second-generation antipsychotics during pregnancy has been shown to increase the risk of gestational diabetes, but this was not limited to a specific trimester.^49^ Some studies have shown that the use of antipsychotics during a pregnancy may increase the risk of maternal diabetes and extrapyramidal symptoms in the mother and malformations, extrapyramidal symptoms and autonomic instability in the infants.^50^ Clinicians did not provide reasons for discontinuation, although it can be speculated that concerns for the well-being of the foetus might have played a role.

Of the mood stabilisers prescribed, current guidance recommends that the use of sodium valproate should be avoided in women of childbearing age because of potential risks of neural tube birth defects and developmental disorders in children who are exposed to it during pregnancy.^32,51,52,53^ Furthermore, women of childbearing age who are currently prescribed sodium valproate should be placed on effective contraception, without stopping or interruption, while on the psychotropic. Important to note that the number of patients receiving sodium valproate in the first trimester was higher than in the second and third trimesters. This may be linked to more than 50% of pregnancies being unplanned, in which case patients often present late in the first trimester on medication that had been prescribed pre-conception. Future studies should explore whether these cases were related to failed contraception or poor contraceptive counselling. Previous studies have also shown that, contrary to national and international guidance on not prescribing sodium valproate to women of childbearing age, this psychotropic continues to be prescribed and that proper counselling is not always provided in South Africa.^54,55,56^

Lamotrigine has been shown to have a similar risk for congenital abnormalities in the offspring of women using this drug compared to women not using anticonvulsant medication during pregnancy; thus, it appears to have a low to minimal risk for foetal structural defects.^57^ Tomson et al.^57^ also concluded that carbamazepine exposure at conception was associated with a risk of major congenital abnormalities, and as such, the first trimester is considered a critical one. Pregnancy planning in conjunction with psychiatric counselling could prevent mothers from conferring risk to their offspring by changing carbamazepine to a safer drug.

The use of topiramate during the first trimester in a study by Hernandez-Diaz et al.^58^ resulted in an increased risk of cleft lip. Studies have also shown an increased risk for autism spectrum disorders and intellectual disability in children with prenatal exposure to topiramate.^59,60^ In our sample, only one patient was prescribed topiramate (diagnosed with anxiety, depression and migraines) and only in the third trimester. The risk of Ebstein’s anomaly has been associated with lithium use in the first trimester.^61^ Given the physiological changes in body water distribution during pregnancy, more regular monitoring of lithium levels during pregnancy is recommended.^28^ In keeping with safety data, lamotrigine was the mood stabiliser of choice in our sample.

Ogawa et al.^62^ found an increased risk of preterm delivery in participants receiving benzodiazepines irrespective of trimester. Given the dependence-producing potential of these drugs, sudden discontinuation is not advisable. Pregnancy-safe medication options and psychotherapy would be more suitable, depending on the patient’s individual circumstances and needs.

The rate of polypharmacy in this study is in line with a similar Australian study by Galbally et al.^63^ (*N* = 535), who found that pregnant women with bipolar mood disorder were twice as likely, compared to all other pregnant women (with a schizophrenia or a non-psychotic severe mental illness primary diagnosis) in the study, to have a prescription of two or more psychotropic agents at any point during their pregnancy. However, Broeks et al.,^42^ using the same definition for polypharmacy as in our study, reported an incidence of polypharmacy double that of our sample, based on data from the Danish National Prescription Registry and investigating psychotropic use among women with bipolar disorder from 12 months pre-conception to 12 months postpartum. Polypharmacy could conceivably increase the risks of psychotropic use during pregnancy *via* an additive effect of the various drugs or through drug–drug interactions.^64^ It was noted by Galbally et al.^63^ that the real-life prescribing pattern of psychotropic medication is not as well researched in comparison with studies that research the effects of a specific medication or medication class on foetal outcomes. The analysis of prescription practice adds a dimension of understanding and can help to develop future studies on psychotropic medication effects during pregnancy.

Ultimately, the potential risks associated with untreated mental illness during pregnancy need to be balanced against the risks of foetal exposure to psychotropics. To assist in determining the safety of general medication use in pregnancy, the US Food and Drug Administration published a ‘Pregnancy and Lactation Labelling Rule’ in 2014.^65^ This is a narrative labelling system that focuses on the pregnancy phase (information obtained from a pregnancy exposure registry), lactation (effect of the drug during breastfeeding) and men and women of reproductive potential (pregnancy testing, contraception and infertility). Psychiatric medication manufacturers are required to review available literature to compile a risk summary for each drug and indicate the limitations of the evidence.^66^ However, compiling a risk statement can be challenging if there is limited data or poor-quality evidence available on the medication. Within this uncertainty, local guidelines, such as the South Africa Department of Health – Standard Treatment Guidelines, can assist health professionals in guiding mental healthcare users and their partners through the treatment options, and health professionals are encouraged to regularly review existing local guidelines on the use of medication in pregnancy.^17,18^

### Limitations

This study described the pattern of medication prescription only; it can therefore not be certain whether the participants used the medication or maintained adherence. The dose of the psychotropic medication was also not indicated, which can have foetal and maternal adverse effects. Some of the medication groupings used were broad, for example, antipsychotic medication; it is possible that nuanced contributions by drugs with dissimilar mechanisms of action are not appropriately highlighted. It was also not possible to explore the context of first trimester prescribing patterns, specifically related to sodium valproate. It would be important to know if patients had been counselled regarding contraception and teratogenic risks associated with pregnancy before starting the medication. Within the antipsychotic group, different medications have different risks to the foetus. Further research is required to determine the long-term effects of exposure to psychotropic medication during pregnancy. The study did not have access to detailed clinical notes from the initial prescribers; therefore, it is unclear whether psychotropic medications were prescribed as a well-motivated exception with the patients’ informed consent (as required) or if these patterns are because of prescribing ignorance, late presentation or drug availability. Of note is that valproate prescription in the first trimester is often a result of patients who have been on valproate before conception and were either not aware that they were pregnant or were not fully informed of the risk.

## Conclusion

In conclusion, the prescription pattern in the study sample appears to be mostly in line with current protocols. The Maudsley Prescribing Guidelines in Psychiatry^36^ recommend that if a woman is planning a pregnancy, one can consider stopping medication if she is at low risk of relapse. If a woman taking psychotropic medication finds out that she is pregnant, the recommendation is that she continue treatment and not switch treatment. Prescribing psychotropic drugs during pregnancy is challenging because of the need to weigh up the potential risks of drug-related effects on both the mother and child against those of discontinuing treatment. A structured approach to patient care and the use of available resources about the effects of individual drugs can aid the clinician who is confronted with such cases.
